# Diagnostic and prognostic value of c-MYC gene expression in hematological malignancies

**DOI:** 10.4314/ahs.v23i2.30

**Published:** 2023-06

**Authors:** Ifeyinwa Maryann Okafor, Henshaw Uchechi Okoroiwu, Christopher Ogar Ogar

**Affiliations:** 1 Department of Haematology and Blood Transfusion Science, Faculty of Medical Laboratory Science, University of Calabar, Nigeria; 2 Department of Medical Laboratory Science, Faculty of Basic Medical Sciences, Arthur Jarvis University, Akpabuyo, Nigeria

**Keywords:** c-MYC, c-MYC screening, prognosis with c-MYC, hematological malignancies, plasma c-MYC, c-MYC in malignancies, c-MYC expression

## Abstract

**Introduction:**

c-MYC plays vital role in regulation of cell proliferation and has been associated with tumorigenesis. This study is aimed at assessing diagnostic and prognostic value of plasma c-MYC expression to aid in early diagnosis and prognosis of hematological malignancies.

**Methods:**

Plasma c-MYC expression was determined by quantitative real time PCR using EVA Green chemistry and cluster of differentiation markers performed via immunocytochemistry.

**Result:**

Plasma c-MYC was higher in subject with hematological malignancies (8.8 ± 1.1) when compared with apparently healthy controls (4.5 ± 0.5). A screening cut-off c-MYC ratio value of 9.42 with sensitivity and specificity of 65.5% and 100% respectively were obtained using receiver operator characteristic curve analysis. Plasma c-MYC was found to have no prognostic value using Kaplan-Meier analysis.

**Conclusion:**

Plasma c-MYC ratio showed promising screening/diagnostic value for hematological malignancies.

## Background

Hematological malignancies refer to a group of neoplastic conditions of lymphoid and hematopoietic tissues which results in leukemia, lymphoma and myelomas 1. The important role of gene expressions in hematological malignancies and cancers in general has become an area of interest in view of diagnosis and prognosis. Nuclear oncogenes which are essential part of cell differentiation, often being pivotal genes in developmental and cell cycle regulation are also implicated in cancer progression 2.

The c-MYC gene is a proto-oncogene located on chromosome 8q24 3. The gene was initially identified as the cellular homolog of the v-MYC oncogene in avian acute leukemia virus in 1978 4,5 c-MYC has been shown over the decades to be an essential global transcriptional factor regulating 10-15% of all human genes 5,6. Under physiological conditions, the main role of c-MYC is to promote cell replication in response to extracellular signals that arouse the cell cycle from rest 7,8,9 Specifically, it is now known to participate in cell functions such as cell cycle, cell growth, cellular metabolism and biosynthesis, differentiation and apoptosis.

In view of this central role in human cell, the expression is tightly regulated at both transcriptional and translational levels 10-14. In hematological malignancies c-MYC regulation is lost and often occurs when it is aberrantly expressed via amplification or genetic alteration, consequently leading to events ranging from promotion of excessive proliferation of altered cells to inhibition of apoptosis 8,15,16. Unlike other proto-oncogens, c-MYC is not activated by oncogenic mutation in the coding sequence. It transforms cells through unregulated over expression of intact c-MYC protein via three basic mechanisms: chromosomal translocation, insertional mutagenesis and gene amplification. 5.

There are several challenges facing the diagnosis and prognosis of hematological malignancies among which include: late presentation of patients in advanced stage of the diseases, financial constraints and wrong/delayed diagnosis owing to poor diagnostic facility such as the use of only morphology for histological diagnosis without immunochemistry and poorly equipped facilities 17. This study is aimed at comparing c-MYC expression in hematological malignancies and apparently healthy subjects as well as assess its diagnostic and prognostic role.

## Methods

### Study design

This study made use of descriptive cross-sectional design with purposive sampling approach.

### Study Area

The study took place at University of Calabar Teaching Hospital, Calabar, Cross River State, Southern Nigeria. The hospital is the major tertiary health institution in the state with 410 beds space: 15 wards and 11 clinics 18. Calabar metropolis in which the hospital is sited has an estimated population of 461,796 19,20,21.

### Data extraction

Demographic information was extracted via questionnaire administered to the study participants.

### Study population and samples

A total of 32 newly diagnosed hematological malignancy patients presenting at Hematology Day care Clinic of the University of Calabar Teaching Hospital, Calabar Nigeria were sampled for analysis of c-MYC gene expression and immunocytochemistry. Twelve apparently healthy control subjects were randomly enrolled. Approximately 5 ml of blood sample was drawn from the antecubital vein of each of the study subjects into ethylenediamine tetra-acetic acid (EDTA) containing tube. Approximately 0.5 ml of the whole blood sample was used in preparing guanidinium isothiocyanate (GITC) trizol lysate for RNA extraction.

### RNA extraction and cDNA synthesis

For each of the sample, 300 µl of the GITC lysate was used for RNA extraction using NorgenBiotek Corp kit (Thoroid Canada), following the manufacturer's instruction. The quality of the RNA samples was assessed using BioRad automated electrophoresis system for 18S/28S ratio and the Eppendorf Bio-photometer Plus for A260/280 ratio. The average 28s/18s RNA ratio was 1.76 (range: 1.60-1.95). The amount of RNA used for cDNA synthesis was 2.0 µg. Random heximer primers (at a final concentration of 300 ng per reaction) from Invitrogen, UK were used for cDNA synthesis with Moloney murine leukemia virus reverse transcriptase and RNasin (RNase inhibitor, Promega, UK). The cDNA synthesis was done immediately after the RNA extraction.

### Real-Time PCR Set-up

EVA Green chemistries were used in the quantitation of c-MYC. The sequences of the primers used are shown in table 1. EVA Green master mix dye (Merck, UK) was used for the c-MYC. The primer was synthesized by IDT, Europe based on Ensembl IDs: ENSE00003475084 and ENSE00003847400. The Ensembl ID of the ABL 1 primer is ENSG00000097007. Relative gene expression levels were calculated by the comparative critical threshold method (2−ΔΔCT) using ABL-1 as a housekeeping gene to normalize expression levels. The housekeeping gene ABL-1 was coamplified with c-MYC on the same plate. The total PCR reaction mix was 20 µL consisting of 1.5 µL primer mix, 10 µL Eva Green master mix, 3.5 µL PCR water and 5 µL cDNA. The thermal profile was as follows: initial denaturation at 95oC for 3 minutes; cycle denaturation at 95^o^C for 15 seconds, annealing at 60^0^C and extension at 72^0^C for 60 and 30 seconds, respectively for 40 cycles. The reactions were carried out on MicPCR Real PCR system (Biomolecular, Australia).

**Table 1:** Primers and probes used in analysis

Gene	Sequence primer 5′-3′		Chromosome location	Amplicon Size, bp
ABL1	Forward	GATACGAAGGGAGGGTGTACCA	9q34	94
	Reverse	CTCGGCCAGGGTGTTGAA		
c-MYC	Forward	CCTACCCTCTCAACGACAGC	8q24	248
	Reverse	c-Myc-R: CTCTGACCTTTTGCCAGGAG		

### Antibodies and Immunocytochemistry

**Figure 1:**
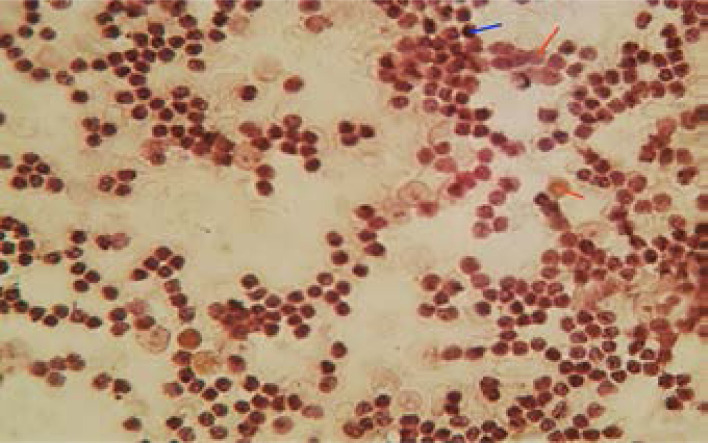
Photomicrograph of immunocytochemistry (Blue line points to positive, red line points to negative)

Immunocytochemistry was performed using reagent kits from Enzo Life Science UK, Polyview plus HRP reagent, (Cat No: Enz-KIT160-0150). The expression of the various cluster of differentiation markers were performed following the manufacturer's instruction. Mouse antibody (primary antibody), POLYVIEW PLUS HRP (anti-mouse), DAB chromogen (chromogen) and HIGHDEF hematoxylin (counter stain) were used. Briefly, the slides were deparaffinized and microwaved for 20 minutes with Citrate buffer pH 6.0 for antigen retrieval. They were immersed in hydrogen peroxidase for 8 minutes before protein blocking for 10 minutes and then incubated in primary antibodies for 3 minutes at room temperature. The detection of immunoreactivity was performed following the manufacturers instruction for mouse and rabbit-specific HRP/DA system. The slides were immersed in ethanol then xylene and mounted DPX mountant and viewed at ×40 and ×100 magnifications. The immunocytochemical staining was semi-quantitatively scored based on the staining characteristics. Dark brown colours indicated positive staining for targets while blues for nuclei was for negative. Cut-off point for positivity was pegged at 30% (Figure 1).

### Statistical analysis

The independent student t-test was used to assess difference between means of two groups (Test vs control; B and non-B malignancies) while one way analysis of variance (ANOVA) was used to assess statistical difference between means of the different types of malignancies. Screening/diagnostic role of the c-MYC expression was performed using Receiver operator characteristics (ROC) curve. The Kaplan-Meier method was used for survival analysis. Overall survival was measured as the time elapsed from the date of diagnosis and enrolment to the date of death from any cause and was only censored from subjects that were confirmed to be alive at the end of the follow up. Groups were compared with Log-rank test. Alpha value was set at 0.05 and all p-value were two tailed. Dot plot was used to represent gene expression levels. The statistic was performed using SPSS (IBM SPSS) version 22.

## Result

### Demographic and clinico-pathological characteristics

**Figure 2:**
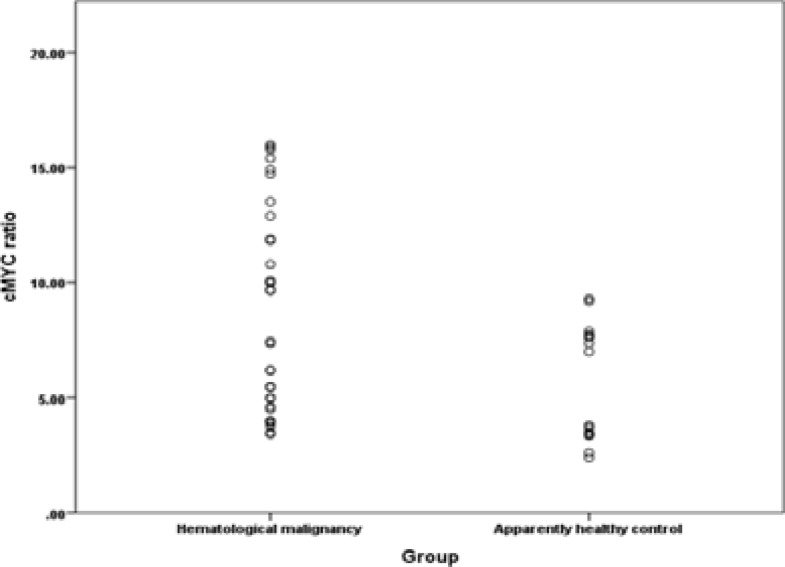
Comparison of Plasma c-MYC expression between subjects with hematological malignancies and that of apparently healthy controls (p=0.038)

**Figure 3:**
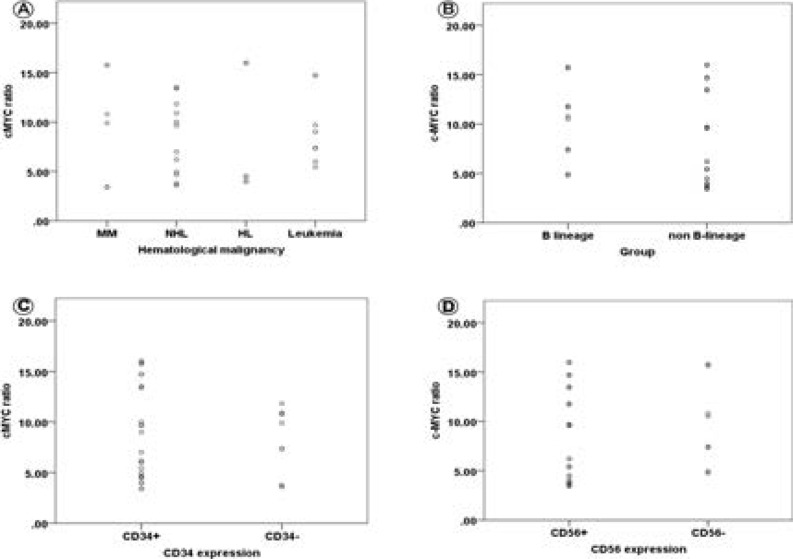
Comparison of Plasma c-MYC expression among some clinico-pathologic variables: (A) Comparison of plasma c-MYC expression based on the different hematological malignancies (p = 0.878); (B) Comparison of plasma c-MYC based on B and non-B lineage (p = 0.457); (C). Comparison of plasma c-MYC based on the expression of CD34 (P = 0.842); (D). Comparison of plasma c-MYC based on the expression of CD56 (p = 0.669)

Thirty two subjects diagnosed of hematological malignancies were enrolled in the study. Demographic and some clinico-pathologic characteristics of the studied subjects are summarized in table 2. In this study, we observed that c-MYC expression (plasma) level in hematological malignancies was significantly higher (8.8 ± 1.1; Mean ± SE) (p<0.05) than that of the apparently healthy control subjects (4.5 ± 0.5; Mean ± SE) (Figure 2). Conversely, we did not find any significant difference (P>0.05) on the level of c-MYC expression based on the type of hematological malignancy as well as B- and non-B lineage stratification. Also, the level of c-MYC expression between cases expressing CD34 and CD56 were comparable (p>0.05) with those not expressing the molecules (Figure 3).

**Table 2:** Demographic and clinical characteristics of the studied subjects

Characteristics	Test subjects	Control subjects
Age (years)	58.5 (20 – 91)	48* (31- 59)
Sex		
Male	24 (75.0)	20 (62.5)
Female	8 (25.0)	12 (37.5)
Hematological malignancy		
Multiple myeloma	6 (18.8)	
Non- Hodgkin's lymphoma	13 (40.6)	
Hodgkin's lymphoma	5 (15.6)	
Leukemia	8 (25.0)	

* : median.

### Receiver operator characteristics (ROC) curve analysis for screening/diagnosis of hematological malignancies

**Figure 4:**
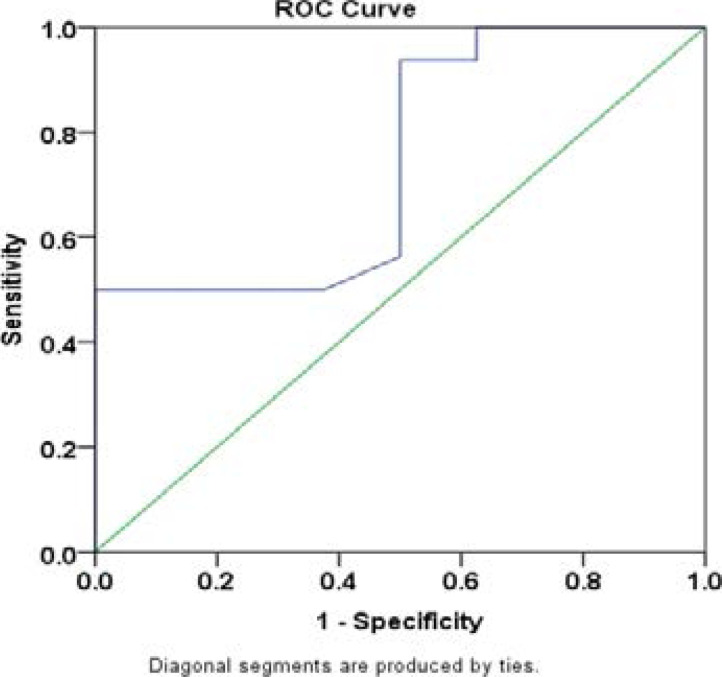
Receiver operator characteristics curve showing the suitability of plasma c-MYC for screening/diagnostic purpose

The ROC curve was plotted and the cut-off value was established via the highest Youden index (sensitivity + specificity -1). Consequently, the maximum Youden J index represented a cut-off value of 9.42 with sensitivity of 65.5% and specificity of 100%. The area under the curve (AUC) was 0.746 (95% CI: (0.533-0.959) (Figure 4).

### Prognostic performance of c-MYC expression/survival analysis

**Figure 5:**
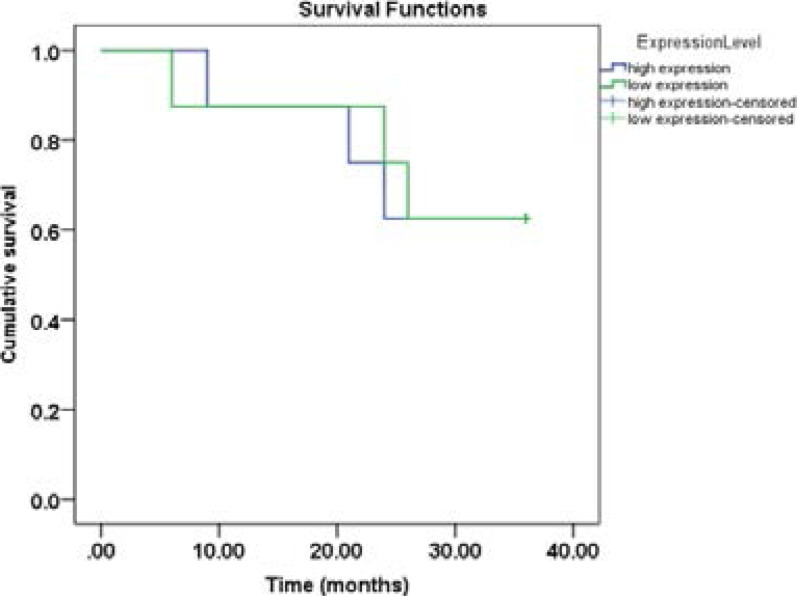
Kaplan-Meier curve showing 3-year disease free survival of high and low c-MYC expression in hematological malignancies Subjects with higher c-MYC expression had comparable worse outcome by disease-free analysis. (p=0.312)

The prognostic factor of disease-free survival of c-MYC expression was evaluated using Kaplan-Meier. The subjects with hematological malignancies were divided into two subgroups (high c-MYC and low c-MYC) using the cut-off value (9.42) generated via ROC curve analysis. The Kaplan-Meier analysis showed that c-MYC expression level was not related to disease free survival. Thus, patients with higher c-MYC and those with lower c-MYC expression had comparable poor outcome (Figure 5).

## Discussion

In this study, we found significantly elevated expression of plasma c-MYC gene in hematological malignancies when compared with those of apparently healthy controls. This finding is consistent with previous report of over expression of c-MYC in Hodgkin's lymphomas 22-24. Aside in hematological malignancies, there have been reports on over expression of c-MYC in breast cancer 25,26. Over expression of c-MYC can occur through a variety of mechanisms among which are: insertional mutagenesis, chromosomal translocation and gene amplification. 27. Stratification of the c-MYC gene expression levels based on B- and non- B lineage showed no significance in both categories.

To ascertain the suitability of c-MYC expression in plasma as a screening test for hematological malignancies, ROC curve was utilized. The area under curve showed a suitability of the model as a screening tool. The high positive predictive potential (100% specificity) considering the low prevalence of hematological malignancies and as well as the cost of gene expression analysis makes it an adequate screening marker for hematological malignancies. More so, the less invasive nature of the analysis (plasma) makes it suitable. Consequently, plasma c-MYC ratio > 9.42 in the presence of other risk factors for hematological malignancies (such as presence of the disease in family, hazardous occupational exposure, etc) is a red flag for possibility of developing hematological malignancies.

The subjects with hematological malignancies were followed up for 3 years in order to determine the prognostic relevance of c-MYC expression. At the end of the follow up, six patients have died. Kaplan-Meier analysis showed that plasma c-MYC level (higher/lower) is not related with shortened three-years disease free survival. This observation is contrary to reports of prognostic relevance of c-MYC protein over expression 28,29. However, it is pertinent to note that the above studies were evaluations of c-MYC protein expression rather than plasma c-MYC gene expression.

However, the present study is prone to a number of limitations. First, we used a small sample size due to the low prevalence of the disease in the locality. A study with larger sample size over time will be required to validate the findings.

## Conclusion

In summary, this study suggests the suitability of plasma c-MYC as a promising screening tool for populations at risk of hematological malignancies.

## List of abbreviations

PCR: polymerase chain reaction
EDTA: ethylenediaminetetracetic acid
GITC: guanidinium isothiocyanate
DNA: Deoxyribonucleic acid
DPX: Dibutylphthalate polystyrene xylene
